# Draft Genome Sequences of Two Bacteria from the *Roseobacter* Group

**DOI:** 10.1128/MRA.00390-21

**Published:** 2021-07-29

**Authors:** Xavier Deogaygay, Nathalie Delherbe, Nicholas J. Shikuma

**Affiliations:** a Department of Biology, San Diego State University, San Diego, California, USA; b Viral Information Institute, San Diego State University, San Diego, California, USA; Indiana University, Bloomington

## Abstract

Here, we report the draft genome sequences of strains HS012 and HS039, which were isolated from cnidarian polyps that had recently undergone metamorphosis. Genomic analyses place these strains within the *Phaeobacter* and *Leisingera* genera, members of the *Roseobacter* group.

## ANNOUNCEMENT

Strains HS012 and HS039 were isolated from artificial seawater containing Hydractinia symbiolongicarpus larvae that had spontaneously undergone metamorphosis to polyps. H. symbiolongicarpus colonies were generously provided by the Nicotra laboratory at the University of Pittsburgh. Strains were isolated from single colonies cultured on artificial seawater-tryptone (ASWT) agar medium (1) at 28°C for 48 h and subsequently were cultured in SWT liquid medium at 28°C for 24 h. Species identity was confirmed by 16S rRNA gene sequencing. Strains were grown in the same manner as for the isolation for DNA extraction.

Genomic DNA was extracted with a Zymo fungal/bacterial DNA miniprep kit and submitted to the Microbial Genome Sequencing Center (MiGS) (Pittsburgh, PA) for Illumina sequencing. The DNA libraries were prepared utilizing a single library preparation method based on the Illumina Nextera kit and were sequenced on the NextSeq 550 platform (2). We obtained paired-end reads (2 × 150 bp) totaling 1,312,670 reads covering a total of 355 Mb for HS012 and 1,817,862 reads covering a total of 473 Mb for HS039. The reads were submitted to the assembly and annotation service at PATRIC v3.6.9 (3) using Trim Galore v0.6.1 (4), Unicycler v0.4.8 (5), and the RAST tool kit (6) for trimming and assembly, with annotation performed via the NCBI Prokaryotic Genome Annotation Pipeline (PGAP) (7). Utilizing the EvalG tool as part of the PATRIC-based assembly (8), the genomes had completeness of 100% and 99.6% for HS012 and HS039, respectively. *Phaeobacter* sp. strain HS012 has a 4.2-Mb genome with a total GC content of 60%, with 26 contigs and an *N*_50_ value of 461,505 bp. *Leisingera* sp. strain HS039 has a 5.0-Mb genome with a total GC content of 62%, with 196 contigs and an *N*_50_ value of 60,120 bp. PATRIC predicted 4,108 and 5,137 coding sequences for HS012 and HS039, respectively. For the software utilized, default parameters were used except where otherwise noted.

**FIG 1:**
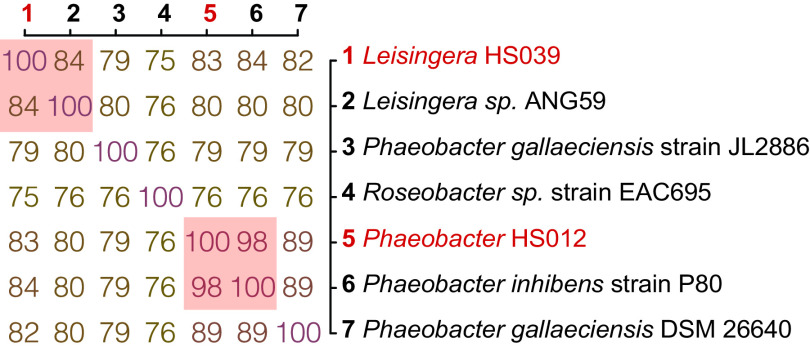
ANI genome-based distance matrix between isolates HS012 and HS039 and members of the *Roseobacter* clade. The ANI genome comparisons were performed using EzBioCloud (10). Numerical values in the matrix indicate percent similarity of nucleotides between the corresponding bacterial strains.

Based on genomic analysis, strain HS012 is closely related to Phaeobacter inhibens strain P80 (9), with an average nucleotide identity (ANI) of 98% (10) (Fig. 1). Strain HS039 is closely related to *Leisingera* sp. strain ANG59, a strain isolated from the accessory nidamental gland of the Hawaiian bobtail squid (Euprymna scolopes) (11), with an ANI of 84% (Fig. 1). These species belong to the *Roseobacter* lineage (*Rhodobacteraceae*), a widespread *Alphaproteobacteria* group (12–16). Because the strains were initially isolated from H. symbiolongicarpus larvae that had spontaneously undergone metamorphosis, we searched the genomes for bacterial genes that had been previously implicated in the stimulation of metamorphosis. Both genomes lack homologs of genes known to induce metamorphosis of tubeworms via metamorphosis-associated contractile structures (17, 18) or corals via tetrabromopyrrole (19). Whether strain HS012 or strain HS039 stimulates H. symbiolongicarpus metamorphosis and produces other yet uncharacterized stimulatory bacterial products will be an interesting direction of future research.

### Data availability.

The genome sequencing and assembly projects for strains HS012 and HS039 in this work have been deposited in GenBank under SRA accession numbers SRR13962962 and SRR13962961, whole-genome sequencing genome accession numbers JAGEUG000000000 and JAGEUH000000000, and raw sequencing SRA accession numbers SAMN18268725 and SAMN18268726, respectively.

## References

[1] Alker AT, Delherbe N, Purdy TN, Moore BS, Shikuma NJ. 2020. Genetic examination of the marine bacterium *Pseudoalteromonas luteoviolacea* and effects of its metamorphosis‐inducing factors. Environ Microbiol 22:4689–4701. doi:10.1111/1462-2920.15211. 32840026 PMC8214333

[2] Baym M, Kryazhimskiy S, Lieberman TD, Chung H, Desai MM, Kishony R. 2015. Inexpensive multiplexed library preparation for megabase-sized genomes. PLoS One 10:e0128036. doi:10.1371/journal.pone.0128036. 26000737 PMC4441430

[3] Davis JJ, Wattam AR, Aziz RK, Brettin T, Butler R, Butler RM, Chlenski P, Conrad N, Dickerman A, Dietrich EM, Gabbard JL, Gerdes S, Guard A, Kenyon RW, MacHi D, Mao C, Murphy-Olson D, Nguyen M, Nordberg EK, Olsen GJ, Olson RD, Overbeek JC, Overbeek R, Parrello B, Pusch GD, Shukla M, Thomas C, Vanoeffelen M, Vonstein V, Warren AS, Xia F, Xie D, Yoo H, Stevens R. 2020. The PATRIC Bioinformatics Resource Center: expanding data and analysis capabilities. Nucleic Acids Res 48:D606–D612. doi:10.1093/nar/gkz943. 31667520 PMC7145515

[4] Krueger F. 2015. Trim Galore: a wrapper tool around Cutadapt and FastQC to consistently apply quality and adapter trimming to FastQ files. https://www.bioinformatics.babraham.ac.uk/projects/trim_galore.

[5] Wick RR, Judd LM, Gorrie CL, Holt KE. 2017. Unicycler: resolving bacterial genome assemblies from short and long sequencing reads. PLoS Comput Biol 13:e1005595. doi:10.1371/journal.pcbi.1005595. 28594827 PMC5481147

[6] Brettin T, Davis JJ, Disz T, Edwards RA, Gerdes S, Olsen GJ, Olson R, Overbeek R, Parrello B, Pusch GD, Shukla M, Thomason JA, Stevens R, Vonstein V, Wattam AR, Xia F. 2015. RASTtk: a modular and extensible implementation of the RAST algorithm for building custom annotation pipelines and annotating batches of genomes. Sci Rep 5:8365–8366. doi:10.1038/srep08365. 25666585 PMC4322359

[7] Tatusova T, Dicuccio M, Badretdin A, Chetvernin V, Nawrocki EP, Zaslavsky L, Lomsadze A, Pruitt KD, Borodovsky M, Ostell J. 2016. NCBI Prokaryotic Genome Annotation Pipeline. Nucleic Acids Res 44:6614–6624. doi:10.1093/nar/gkw569. 27342282 PMC5001611

[8] Parks DH, Imelfort M, Skennerton CT, Hugenholtz P, Tyson GW. 2015. CheckM: assessing the quality of microbial genomes recovered from isolates, single cells, and metagenomes. Genome Res 25:1043–1055. doi:10.1101/gr.186072.114. 25977477 PMC4484387

[9] Kopejtka K, Lin Y, Jakubovičová M, Koblížek M, Tomasch J. 2019. Clustered core- and pan-genome content on *Rhodobacteraceae* chromosomes. Genome Biol Evol 11:2208–2217. doi:10.1093/gbe/evz138. 31273387 PMC6699656

[10] Yoon SH, Ha SM, Kwon S, Lim J, Kim Y, Seo H, Chun J. 2017. Introducing EzBioCloud: a taxonomically united database of 16S rRNA gene sequences and whole-genome assemblies. Int J Syst Evol Microbiol 67:1613–1617. doi:10.1099/ijsem.0.001755. 28005526 PMC5563544

[11] Suria AM, Tan KC, Kerwin AH, Gitzel L, Abini-Agbomson L, Bertenshaw JM, Sewell J, Nyholm SV, Balunas MJ. 2020. Hawaiian bobtail squid symbionts inhibit marine bacteria via production of specialized metabolites, including new bromoalterochromides BAC-D/D′. mSphere 5:e00166-20. doi:10.1128/mSphere.00166-20. 32611694 PMC7333567

[12] Majzoub ME, McElroy K, Maczka M, Thomas T, Egan S. 2018. Causes and consequences of a variant strain of *Phaeobacter inhibens* with reduced competition. Front Microbiol 9:2601. doi:10.3389/fmicb.2018.02601. 30450086 PMC6224355

[13] Martens T, Heidorn T, Pukal R, Simon M, Tindall BJ, Brinkhoff T. 2006. Reclassification of *Roseobacter gallaeciensis* Ruiz-Ponte et al. 1998 as *Phaeobacter gallaeciensis* gen. nov., comb. nov., description of *Phaeobacter inhibens* sp. nov., reclassification of *Ruegeria algicola* (Lafay et al. 1995) Uchino et al. 1999 as *Marinovum algicola* gen. nov., comb. nov., and emended descriptions of the genera *Roseobacter*, *Ruegeria* and *Leisingera*. Int J Syst Evol Microbiol 56:1293–1304. doi:10.1099/ijs.0.63724-0. 16738106

[14] Cavalcanti GS, Wasserscheid J, Dewar K, Shikuma NJ. 2020. Complete genome sequences of two marine biofilm isolates, *Leisingera* sp. nov. strains 201A and 204H, novel representatives of the *Roseobacter* group. Microbiol Resour Announc 9:e00505-20. doi:10.1128/MRA.00505-20. 32646902 PMC7348020

[15] Lau SCK, Mak KKW, Chen F, Qian P-Y. 2002. Bioactivity of bacterial strains isolated from marine biofilms in Hong Kong waters for the induction of larval settlement in the marine polychaete *Hydroides elegans*. Mar Ecol Prog Ser 226:301–310. doi:10.3354/meps226301.

[16] Luo H, Moran MA. 2014. Evolutionary ecology of the marine *Roseobacter* clade. Microbiol Mol Biol Rev 78:573–587. doi:10.1128/MMBR.00020-14. 25428935 PMC4248658

[17] Ericson CF, Eisenstein F, Medeiros JM, Malter KE, Cavalcanti GS, Zeller RW, Newman DK, Pilhofer M, Shikuma NJ. 2019. A contractile injection system stimulates tubeworm metamorphosis by translocating a proteinaceous effector. Elife 8:e46845. doi:10.7554/eLife.46845. 31526475 PMC6748791

[18] Shikuma NJ, Pilhofer M, Weiss GL, Hadfield MG, Jensen GJ, Newman DK. 2014. Marine tubeworm metamorphosis induced by arrays of bacterial phage tail-like structures. Science 343:529–533. doi:10.1126/science.1246794. 24407482 PMC4949041

[19] El Gamal A, Agarwal V, Diethelm S, Rahman I, Schorn MA, Sneed JM, Louie GV, Whalen KE, Mincer TJ, Noel JP, Paul VJ, Moore BS, El Gamal A, Agarwal V, Diethelm S, Rahman I, Schorn MA, Sneed JM, Louie GV, Whalen KE, Mincer TJ, Noel JP, Paul VJ, Moore BS. 2016. Biosynthesis of coral settlement cue tetrabromopyrrole in marine bacteria by a uniquely adapted brominase- thioesterase enzyme pair. Proc Natl Acad Sci U S A 113:3797–3802. doi:10.1073/pnas.1519695113. 27001835 PMC4833250

